# Editorial: Maternal substance and alcohol use and contextual issues

**DOI:** 10.3389/fpsyt.2024.1432117

**Published:** 2024-07-23

**Authors:** Yukiko Washio, Petal Petersen Williams, Krystyna R. Isaacs

**Affiliations:** ^1^ Substance Use, Gender and Applied Research, RTI (Research Triangle Institute) International, Research Triangle Park, NC, United States; ^2^ Department of Obstetrics, Gynecology and Reproductive Sciences, Temple University Lewis Katz School of Medicine, Philadelphia, PA, United States; ^3^ Mental Health, Alcohol, Substance Use and Tobacco Research Unit, South African Medical Research Council, Cape Town, South Africa; ^4^ Department of Health Sciences, University of York, York, United Kingdom; ^5^ Department of Global Health, Institute for Life Course Health Research, Stellenbosch University, Cape Town, South Africa; ^6^ SciConsult Solutions, Coppell, TX, United States

**Keywords:** maternal, infant, substance and alcohol use, contextual, perinatal

Recent research advancements have delineated the impact of perinatal substance and alcohol use on the health of mothers and fetuses/infants (1–3) and also the bidirectional impact between perinatal substance and alcohol use and contextual issues (4–10). Adverse effects of perinatal substance and alcohol use include miscarriage, intrauterine growth restriction, low birth weight, shorter gestational weeks, increases in NICU admission, and stillbirth (1, 11–15). Recent evidence also identified independent adverse effects of breastfeeding while using alcohol on infant neurocognitive and physical development (16). This is concerning given the fact that even among people who stop drinking during pregnancy, many will return to drinking post-partum. A paucity of evidence about drinking during breastfeeding and few recommendations contribute to this phenomenon (17).

When perinatal substance and alcohol use occurs, there are often co-occurring psychosocially and culturally relevant contextual issues, such as socioeconomic disadvantage, lack of social support, trauma exposure, and depression (18–20). Given the significant involvement of perinatal substance and alcohol use in compromised sexual health (7, 21), these intertwined physical and mental health issues are clustered as a SAVA syndemic (substance abuse, violence, and AIDS) (22).

While using a single substance or alcohol during perinatal periods leads to pregnancy, birth, and infant complications (1, 2, 23–27), combined use of substances and alcohol use could further exacerbate their adverse effects on maternal-infant health (18–20, 28, 29). Because of continued cannabis legalization (30), an increase in perinatal cannabis use is a concern (31) (25, 26). The consequences of legalizing cannabis need to be delineated for pregnant and breastfeeding people (32–35).

The opioid epidemic in the United States (US) has led to a significant increase in perinatal opioid use (36). On top of this, the prevalence of overall perinatal substance and alcohol use as well as relevant contextual issues including violence exposure and maternal depression and anxiety has worsened since the global COVID-19 pandemic (37–42).

The current Research Topic, Maternal Substance and Alcohol Use and Contextual Issues, therefore focused on a set of qualitative and quantitative studies on maternal substance and alcohol use and associated contextual issues, with the goal of proposing new approaches to provide evidence-based information and treatment interventions. This Research Topic demonstrates a diversity in study settings, types of studies, and topic focus. The settings include Poland, South Africa, and the Philippines, in addition to the United States. The studies included both qualitative and quantitative research, and ranged from pilot and usability testing studies, to systematic reviews, to survey research. The topics included prenatal substance and alcohol use, stigma and mental health issues among pregnant and parenting people, and postpartum smoking relapse and substance use.

Two qualitative studies from South Africa (Petersen Williams et al., Petersen Williams et al.) investigated the development of interventions to encourage alcohol abstinence during pregnancy and breastfeeding. In both studies, the need for an alcohol intervention program was highlighted and informed the adaptation process for interventions that are culturally relevant and acceptable to the needs of the local context. Another study (Nguyen et al.) examined knowledge, attitudes, practices, and beliefs regarding prenatal alcohol consumption. Of particular interest in this study conducted in the Philippines (Huang et al.) was the widespread consumption (75%) of a local alcoholic beverage during pregnancy, which was believed to not contain alcohol and, in some instances, even fed to infants. Encouragingly, nearly all mothers (98%) were willing to reduce consumption when told that the practice negatively impacts pregnancies. An intervention study in Poland (Okulicz-Kozaryn et al.) aimed to reduce the risk of prenatal alcohol exposure in the general population of women of childbearing age including reduction of risky alcohol consumption, increasing effective contraception use, and increasing use of professional support to address the complex psychological, medical and social challenges which may increase risk of alcohol use during pregnancy. Follow-up data indicated that risky alcohol consumption dropped by 81%; contraception use increased by 15% and visiting a gynecologist increased by 39%. The most prominent changes were observed in the moderate-risk group.

Opioid use disorder is a leading cause of pregnancy-associated deaths. One study in the United States (Nguyen et al.) found that among patients who were incarcerated and initiated buprenorphine (BUP) treatment, the majority (97%) remained on BUP at delivery compared to those who were not incarcerated at BUP initiation (79%). Pregnant and parenting women recovering from substance use disorder (SUD) are at risk of insufficient recovery support. Another US study (Isaacs et al.) tested the usability and acceptability of a Plan of Safe Care (POSC) platform which combined a mobile health app with a web-based case management system. Family services staff, treatment center staff, and mothers with SUD rated the platform as usable and acceptable. A qualitative study (Young-Wolff et al.) in the US found that coping with mental health symptoms and stress were identified as drivers of perceived COVID-19 pandemic-related increases in prenatal cannabis use in 2021.

Researchers in the US evaluated the delivery of attentional retraining (AR) for smoking cues in perinatal smokers, also utilizing a mobile intervention (Forray et al.). They found evidence that AR reduced attentional bias compared with controls but found no evidence that AR reduced craving or smoking during the study period.

Some of the contextual issues to do with maternal substance use were also addressed in the Research Topic. In a systematic review and meta-analysis (Pacho et al.), the authors provided evidence of an increased risk of postpartum depression among pregnant substance users, and this was particularly the case for those using multiple substances or tobacco.

Stigma remains a huge barrier to receiving care for SUD, particularly among pregnant and parenting people. Another US study (Lipsett et al.) explored stigma reduction practices within the research community that can increase the uptake of evidence-based treatment programs and proposed six strategies for this to happen.

The collection of these publications gives us a glimpse of what is known about maternal substance and alcohol use and relevant contextual issues and what are the future research directions that subsequent studies need to follow.

## Author contributions

YW: Conceptualization, Writing – original draft, Writing – review & editing. PP: Conceptualization, Writing – review & editing. KI: Writing – review & editing.
